# Tamoxifen as an effective neuroprotectant against early brain injury and learning deficits induced by subarachnoid hemorrhage: possible involvement of inflammatory signaling

**DOI:** 10.1186/1742-2094-10-157

**Published:** 2013-12-28

**Authors:** Xuebo Sun, Chengyuan Ji, Tong Hu, Zhong Wang, Gang Chen

**Affiliations:** 1 Department of Neurosurgery, The First Affiliated Hospital of Soochow University, 188 Shizi Street, Suzhou 215006, Jiangsu Province, China; 2 Institute of Neuroscience, Soochow University, 199 Renai Road, Suzhou Jiangsu Province, China

**Keywords:** Tamoxifen, Early brain injury, Learning deficits, Subarachnoid hemorrhage, Inflammation

## Abstract

**Background:**

Tamoxifen, a selective estrogen receptor modulator, has successfully been used to treat several animal models of brain injury, but the underlying mechanisms remain unclear. This study was undertaken to evaluate the effect of tamoxifen on the toll-like receptor 4 (TLR4)- and nuclear factor-κB (NF-κB)-related inflammatory signaling pathway and secondary brain injury in rats after subarachnoid hemorrhage (SAH).

**Methods:**

Adult male Sprague-Dawley rats were divided into four groups: (1) control group (n = 28); (2) SAH group (n = 28); (3) SAH + vehicle group (n = 28); and (4) SAH + tamoxifen group (n = 28). All SAH animals were subjected to injection of autologous blood into the prechiasmatic cistern once on day 0. In SAH + tamoxifen group, tamoxifen was administered intraperitoneally at a dose of 5 mg/kg at 2 h, 12 h, and 36 h after SAH. In the first set of experiments, brain samples were extracted and evaluated at 48 h after SAH. In the second set of experiments, the Morris water maze was used to investigate cognitive and memory changes.

**Results:**

We found that treatment with tamoxifen markedly inhibited the protein expressions of TLR4, NF-κB and the downstream inflammatory agents, such as interleukin-1β (IL-1β), tumor necrosis factor-α (TNF-α), interleukin-6 (IL-6), and intercellular adhesion molecule-1 (ICAM-1). Administration of tamoxifen following SAH significantly ameliorated the early brain injury (EBI), such as brain edema, blood-brain barrier (BBB) impairment, and clinical behavior scale. Learning deficits induced by SAH were markedly alleviated after tamoxifen treatment.

**Conclusions:**

Post-SAH tamoxifen administration may attenuate TLR4/NF-kappaB-mediated inflammatory response in the rat brain and result in abatement of the development of EBI and cognitive dysfunction after SAH.

## Introduction

In China, stroke is the third leading cause of death, approximately 20% of which are due to aneurysmal subarachnoid hemorrhage (SAH) [1]. In the past three decades, there are far more studies focusing on vasospasm of the cerebral arteries than on the pathophysiological changes in the brain after SAH, which also could lead to disastrous outcomes [2]. Early brain injury (EBI) and secondary cognitive or neurobehavioral dysfunction after SAH have been well documented, but the underlying mechanisms still remain unclear [3]. On the one hand, EBI is the most common cause of disability and mortality in patients suffering from SAH. Treatment of EBI is considered a major goal in the management of SAH patients. However, the exact molecular mechanism of EBI still remains unknown, which has hindered the development of effective and specific treatment paradigms for EBI [4]. On the other hand, approximately 50% of all SAH patients die from EBI, and many of those who do survive have lasting cognitive deficits [5]. Nowadays, treatment of cognitive dysfunction has also been considered as a major target in the management of patients surviving cerebral aneurysm rupture.

Tamoxifen is an antagonist of the estrogen receptor in breast tissue via its active metabolite, hydroxytamoxifen. In other tissues, it behaves as an agonist, and thus may be characterized as a mixed agonist/antagonist [6]. Tamoxifen is the usual endocrine (anti-estrogen) therapy for hormone receptor-positive breast cancer in premenopausal women, and is also a standard in postmenopausal women. Several previous reports from experimental studies have demonstrated that tamoxifen plays neuroprotective roles in spinal cord injury [7], intracerebral hemorrhage [8], brain ischemia [9], and hypoxic-ischemic brain injury [10]. At the same time, previous studies have proven that tamoxifen could induce an anti-inflammatory response in acute models of mouse and rat microglial cells; this response seemed not to be estrogen receptor-mediated but probably was attributable to some tamoxifen-induced modulation of pro-inflammatory signaling cascades [11,12].

Nevertheless, until now it was unknown whether tamoxifen could influence TLR4/NF-κB-related pro-inflammatory pathways in the brain and decrease the severity of brain injury after SAH. Thus, the aim of the current study was to determine whether tamoxifen could attenuate the SAH-induced activation of the TLR4/NF-κB signaling pathway in the cortex and promote neurological function and behavioral recovery after SAH.

## Materials and methods

### Animals

Male Sprague-Dawley (SD) rats (310 to 360 g; 12 weeks old) were purchased from Animal Center of the Chinese Academy of Sciences, Shanghai, China. The rats were housed in temperature- and humidity-controlled animal quarters with a 12-h light/dark cycle. All procedures were approved by the Institutional Animal Care Committee of Soochow University and were in accordance with the guidelines of the National Institutes of Health on the care and use of animals.

Following intraperitoneal anesthesia of an animal with urethane (1,000 mg/kg), the animal’s head was fixed in the stereotactic frame. The body temperature was maintained at 37.5 ± 0.5°C with an automatic heating pad (LSI Letica Scientific Instruments, Barcelona, Spain). The tail artery was cannulated to measure mean arterial blood pressure (MABP) and to obtain blood samples. The animals were placed in a stereotaxic frame (David Kopf Instruments, Tujunga, CA, USA) with the mouthpiece at 0°C. A laser Doppler flowmeter (LDF) (MBF3D, Moor Instruments, Millwey, Axminster, Devon, UK) was used for continuous monitoring of cerebral blood flow (CBF) in the area of the cerebral cortex supplied by the middle cerebral artery (MCA). To enable placement of the LDF probes, a bur hole was drilled 5 mm left lateral and 1 mm posterior to the bregma without injury to the dura mater.

### Rat subarachnoid hemorrhage model

The experimental SAH model was produced using stereotaxic insertion of a needle with a rounded tip and a side hole into the prechiasmatic cistern according to our previous study [13]. The amount of 0.3 ml non-heparinized fresh autologous arterial blood was slowly injected into the prechiasmatic cistern for 20 s with a syringe pump under aseptic technique. Control animals were injected with 0.3 ml saline. The animals were allowed to recover 45 min after SAH. After operation procedures, the rats were then returned to their cages and the room temperature kept at 23 ± 1°C. Twenty milliliters of 0.9% NaCl was injected subcutaneously right after the operation to prevent dehydration. Heart rate and rectal temperature were monitored, and the rectal temperature was kept at 37 ± 0.5°C by using physical cooling (ice bag) when required, throughout experiments. In the present study, it was observed that the inferior basal temporal lobe was always stained by blood. The tissue of the cortex was separated on ice while being viewed under a microscope and was frozen in liquid nitrogen immediately for molecular biological and biochemical experiments.

### Experimental design

**Figure 1:**
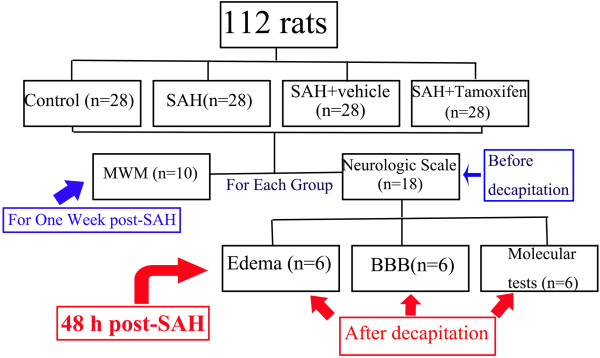
Experimental design.

As shown in Figure 1, we established four experimental groups in a randomized fashion: (a) the control group (n = 28), (b) the SAH group (n = 28), (c) the SAH + vehicle group (n = 28), and (d) the SAH + tamoxifen group (n = 28). Rats of the SAH + tamoxifen group received 5 mg/kg tamoxifen intraperitoneally (i.p.) at 2, 12, and 36 h after the surgery. Rats of the SAH + vehicle group received equal volumes of 2-hydroxypropyl-β-cyclodextrin with the same schedule. In the first experimental setting, the animals were decapitated 48 h after injury for tissue assays. In the second experiment, the animals were trained and evaluated in a Morris water maze (MWM). The dose was chosen according to Xie *et al*. [8] since they observed beneficial effects on improving neurological function and reducing brain edema in the intracerebral hemorrhage model.

### Brain water content

Brain edema was determined using the wet/dry method as previously described where percent brain water = ((wet weight - dry weight) /wet weight) × 100% [14]. Briefly, brains were rapidly removed from the skull and the bilateral brains were separated. Both were placed separately into pre-weighed and labeled glass vials and weighed. The vials were then placed in an oven for 72 h at 100°C and then re-weighed to obtain dry weight content. The number of animals used in each group for brain edema study was control (n = 6), SAH (n = 6), SAH + vehicle (n = 6) and SAH + tamoxifen (n = 6).

### Blood-brain barrier permeability

Blood-brain barrier (BBB) permeability was determined by Evans blue (EB) extravasation at 48 h after SAH. Briefly, 2% Evans blue was injected intravenously at a dose of 2 ml/kg. Animals were then re-anesthetized after 1 h with urethane (1,000 mg/kg) and perfused using saline to remove intravascular EB dye. Animals were then decapitated, and the entire brain of each was removed and homogenized in phosphate buffered saline. Trichloroacetic acid was then added to precipitate protein, and the samples were cooled and centrifuged. The resulting supernatant was measured for absorbance of EB at 610 nm using a spectrophotometer. The number of animals used in each group for the BBB permeability study was control (n = 6), SAH (n = 6), SAH + vehicle (n = 6) and SAH + tamoxifen (n = 6).

### Neurologic scoring

Three behavioral activity examinations (Table 1) were performed at 48 h after SAH using the scoring system reported previously to record appetite, activity and neurological deficits [15]. The number of animals used in each group for neurologic scoring study was control (n = 18), SAH (n = 18), SAH + vehicle (n = 18) and SAH + tamoxifen (n = 18).

**Table 1:** Behavior and activity scores

**Category**	**Behavior**	**Score**
Appetite	Finished meal	0
	Left meal unfinished	1
	Scarcely ate	2
Activity	Walk and reach at least three corners of the cage	0
	Walk with some stimulation	1
	Almost always lying down	2
Deficits	No deficits	0
	Unstable walk	1
	Impossible to walk	2

### Western blot analysis

The frozen brain tissue was mechanically lysed in 20 mM Tris, pH 7.6, which contains 0.2% SDS, 1% Triton X-100, 1% deoxycholate, 1 mM phenylmethylsulphonyl fluoride (PMSF), and 0.11 IU/ml aprotinin (all purchased from Sigma-Aldrich, Inc., St. Luis, MO, USA). Lysates were centrifuged at 12,000 × g for 20 min at 4°C. The protein concentration was estimated by the Bradford method using the Nanjing Jiancheng (NJJC) protein assay kit (Nanjing Jiancheng Bioengineering Institute, Nanjing, China). The samples (60 μg per lane) were separated by 8% SDS-PAGE and electro-transferred onto a polyvinylidene-difluoride (PVDF) membrane (Bio-Rad Laboratories, Hercules, CA, USA). The membrane was blocked with 5% skimmed milk for 2 h at room temperature, incubated overnight at 4°C with primary antibodies directed against the TLR4, NF-κB P50, and ICAM-1 (all from Santa Cruz Biotechnology, Inc., Santa Cruz, CA, USA) in PBS + Tween 20 (PBST) at dilutions of 1:200, and β-tubulin (diluted 1:8000 in PBST, Sigma-Aldrich, Inc., St. Luis, MO, USA) was used as a loading control. We detected TLR4 at 95 kDa, NF-κB at 50 kDa, ICAM-1 at 60 kDa, and β-tubulin at 50 kDa. After the membrane was washed for 10 min each for six times in PBST, it was incubated in the appropriate HRP-conjugated secondary antibody (diluted 1:400 in PBST) for 2 h. The blotted protein bands were visualized by enhanced chemiluminescence (ECL) Western blot detection reagents (Amersham, Arlington Heights, IL, USA) and were exposed to X-ray film. Developed films were digitized using an Epson Perfection 2480 scanner (Seiko Corp, Nagano, Japan). Optical densities were obtained using Glyko Bandscan software (Glyko, Novato, CA, USA) and the protein expression levels were normalized to β-tubulin.

### Nuclear protein extract and electrophoretic mobility shift assay

Nuclear protein was extracted and quantified as described [16]. Electrophoretic mobility shift assay (EMSA) was performed using a commercial kit (Gel Shift Assay System; Promega, Madison, WI, USA) following the methods in our laboratory. The NF-κB oligonucleotide probe (5’-AGTTGAGGGGACTTTCCCAGGC-3’) was end-labeled with [γ-32P]ATP (Free Biotech, Beijing, China). EMSA was performed according to our previous study [16].

### Enzyme-linked immunosorbent assay

The levels of inflammatory mediators were quantified using specific ELISA kits for rats according to the manufacturers' instructions (TNF-α from Diaclone Research, Besançon, France; IL-1β, IL-6 from Biosource Europe SA, Nivelles, Belgium) and our previous study [16]. Values were expressed as ng/g protein.

### Behavior testing

Spatial learning and memory were assessed using a modified version of the Morris water maze (MWM) including cued learning procedure, spatial acquisition task, reference memory task, and working memory task according to the previous study [17]. A camera mounted in the center of the ceiling above the pool tracked the rat (Poly Track System, San Diego Instruments, San Diego, CA, USA). Behavior testing was performed between 10:00 and 18:00. All animals were housed at a constant temperature of 22°C, under a 12-h light/dark cycle (light switched on at 6:00 AM), with free access to food and water. The number of animals used in each group for MWM study was control (n = 10), SAH (n = 10), SAH + vehicle (n = 10) and SAH + tamoxifen (n = 10).

### Experiments for tamoxifen study in control rats

For evaluating the effects of tamoxifen in control rats, we established two experimental groups in a randomized fashion: the control + vehicle group (n = 8) and the control + tamoxifen group (n = 8). Rats of control + tamoxifen group received 5 mg/kg tamoxifen i.p. at 2, 12, and 36 h after the sham surgery. Rats of control + vehicle group received equal volumes of 2-hydroxypropyl-β-cyclodextrin with the same schedule. The animals were decapitated 48 h after sham injury for tissue assays.

### Statistical analysis

All data were presented as mean ± SD. SPSS 12.0 was used for statistical analysis of the data. All data were subjected to a one-way ANOVA. Differences between experimental groups were determined by Fisher’s LSD post-test. Statistical significance was inferred at *P* <0.05.

## Results

### General observation

**Figure 2:**
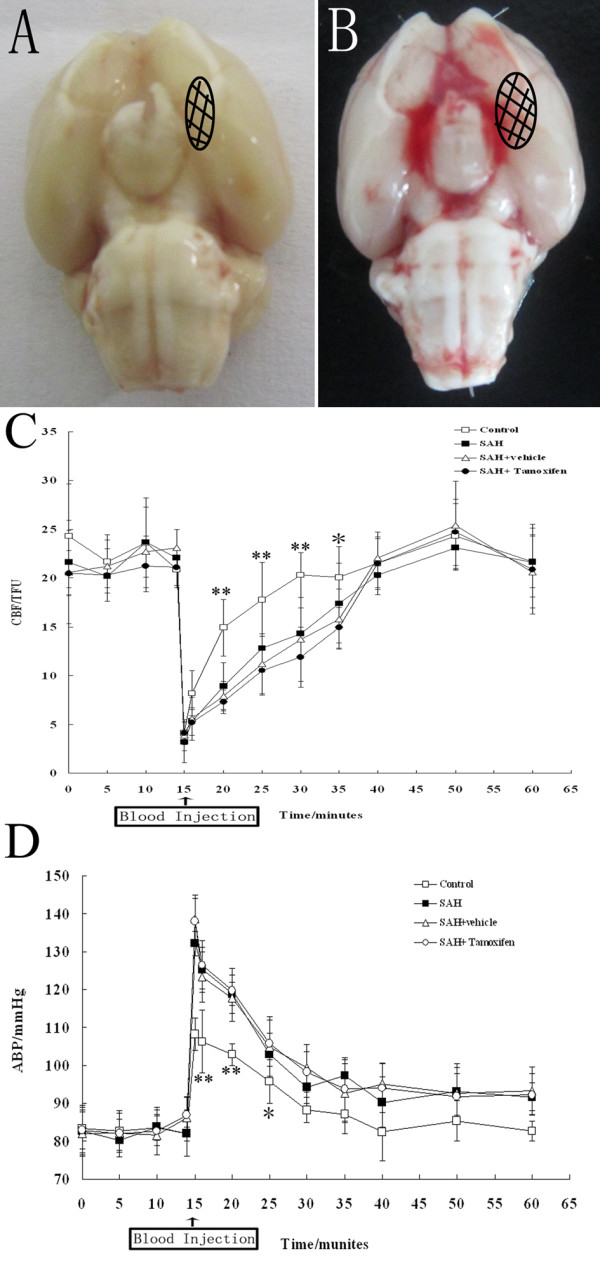
**Schematic representation of the analyzed area induced by subarachnoid hemorrhage (SAH). (A)** control group and **(B)** SAH group. **C** and **D**: The time course of cerebral blood flow (CBF) and mean arterial blood pressure (MABP) in control group (n = 18), SAH group (n = 18), SAH + vehicle group (n = 18), and SAH + tamoxifen group (n = 18). **(C)** Graphs of CBF in middle cerebral artery (MCA) area measured by continuous laser Doppler flowmeter over the left hemisphere. **(D)** Secondary to injection of saline or blood, MABP increased sharply in all four groups. *P <0.05 and **P <0.01 between control animals versus SAH animals.

No significant changes in body weight, MABP, temperature, or injected arterial blood gas data were detected in any of the experimental groups (data not shown). The mortality rate of rats in the control group was 0% (0/28 rats), and it was 20% (21/105 rats) in the SAH group. As shown in Figure 2A, the rats in SAH groups exhibited blood clots over the basal surface of the brainstem and Willis circle. The data of CBP and MABP are shown in Figure 2B and 2C. In SAH group, CBF decreased from 23.1 ± 1.6 to 3.7 ± 1.1 TFU within 20 s during the blood injection, and then increased to the baseline in 25 min (Figure 2B). After SAH, MABP increased immediately from 83.4 ± 5.3 to 134.5 ± 4.1 mmHg, but returned to values of baseline within 15 min (Figure 2C).

### Tamoxifen ameliorated early brain injury after experimental subarachnoid hemorrhage

**Figure 3:**
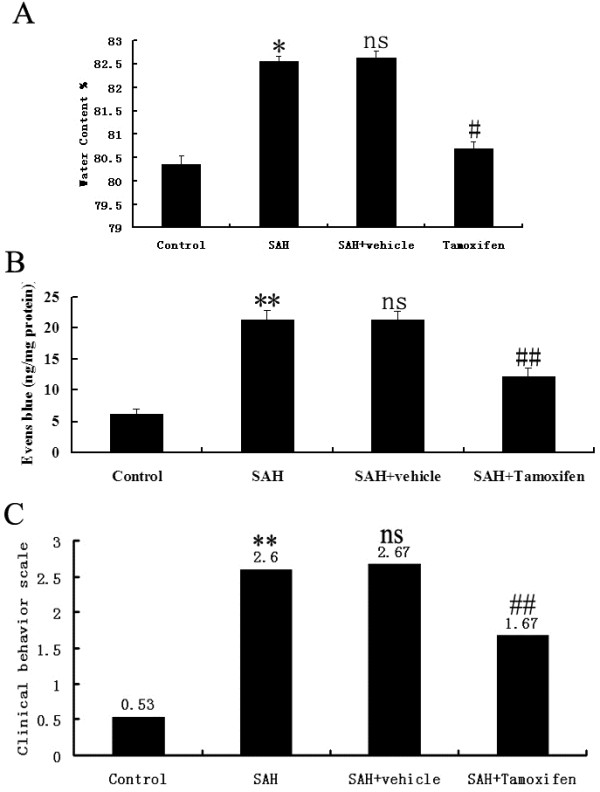
**Alterations in brain water content in control group (n = 6), subarachnoid hemorrhage** (**SAH) group (n = 6), SAH + vehicle group (n = 6), and SAH + tamoxifen group (n = 6). (A)** The brain water content was increased significantly at 48 h after SAH. Tamoxifen treatment markedly reduced brain water content. **(B)** Alterations in Evans blue extravasation in control group (n = 6), SAH group (n = 6), SAH + vehicle group (n = 6), and SAH + tamoxifen group (n = 6). SAH could induce a marked increase of blood-brain barrier (BBB) extravasation in the rat brain compared with control group. After tamoxifen administration, the Evans blue extravasation was significantly reduced as compared with SAH + vehicle group. **(C)** Effects of tamoxifen administration on functional outcomes in the control group (n = 18), SAH group (n = 18), SAH + vehicle group (n = 18), and SAH + tamoxifen group (n = 18). Compared to rats in SAH + vehicle group, Tamoxifen administration attenuated the SAH-induced impairment in the performance tested at 48 h after SAH. *P <0.05 and **P <0.01 between control animals versus SAH animals; #*P* <0.05 and ##*P* <0.01 between SAH + vehicle animals versus SAH + tamoxifen animals; n.s. *P* >0.05 between SAH animals versus SAH + vehicle animals.

A significant increase (*P* <0.05) in water content was detected in the brain samples of injured side at 48 h after SAH when compared with rats in control group (Figure 3A). The mean value of brain water content in the brain was decreased by tamoxifen administration (*P* <0.05) as compared with SAH + vehicle group. The pattern of Evans blue extravasation following SAH is shown in Figure 3B. Rats in SAH and SAH + vehicle groups demonstrated a significant increase (*P* <0.01) in BBB permeability to Evans blue relative to rats of control group. Administration of tamoxifen significantly inhibited Evans blue extravasation (*P* <0.01), indicating a reduced BBB opening in response to tamoxifen treatment. As compared with the control group, clinical behavior function impairment caused by SAH was evident in SAH subjects (*P* <0.01, Figure 3C). No significant difference was seen between the SAH group and SAH + vehicle group (*P* >0.05). Tamoxifen treated rats showed better performance in this scale system than vehicle-treated rats at 48 h after SAH (Figure 3C), and the difference was statistically significant (*P* <0.01).

### Western blot analysis for detecting TLR4, NF-κB, and ICAM-1 expressions after subarachnoid hemorrhage

**Figure 4:**
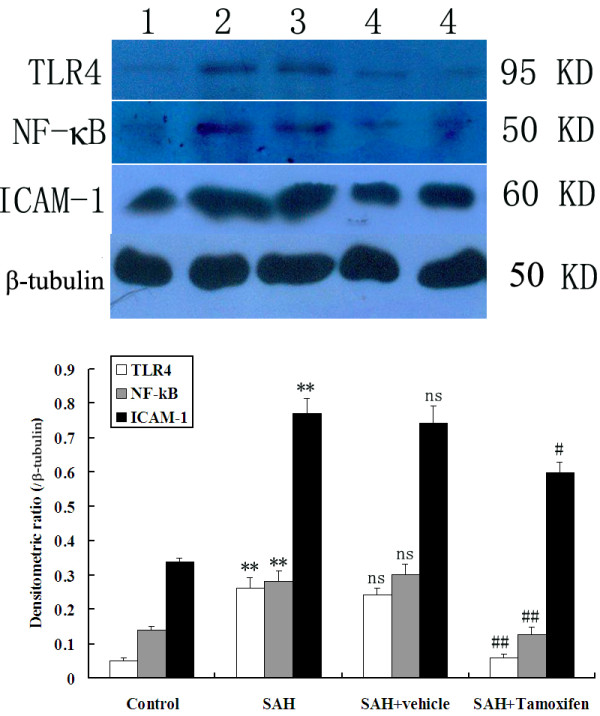
**Representative autoradiogram of TLR4, NF-κB, and ICAM-1 expression in the brain after subarachnoid hemorrhage (SAH).** Upper: We detected TLR4 at 95 kDa, NF-κB at 50 kDa, ICAM-1 at 60 kDa, and the loading control β-tubulin at 50 kDa. It shows that the expression of these proteins was increased in the SAH groups and downregulated after tamoxifen treatment. Lane 1, control; lane 2, SAH; lane 3, SAH + vehicle; lanes 4, SAH + tamoxifen, respectively. Bottom: Quantitative analysis of the Western blot results shows that these protein levels in SAH groups are significantly higher than in the control group and were inhibited by tamoxifen. Bars represent the mean ± SD (n = 6, each group). **P <0.01 between control animals versus SAH animals; #*P* <0.05 and ##*P* <0.01 between SAH + vehicle animals versus SAH + tamoxifen animals; n.s. *P* >0.05 between SAH animals versus SAH + vehicle animals.

The protein levels of TLR4, NF-κB, and ICAM-1 were detected by western blot. These proteins were expressed at a low level in the rat brains of control group. The levels of TLR4, NF-κB, and ICAM-1 were significantly increased in the cortex in SAH group as compared with that of control group (*P* <0.05). The protein expressions had no significant difference between SAH group and SAH + vehicle group (*P* >0.05). The expressions of TLR4, NF-κB, and ICAM-1 in the brains of SAH + Tamoxifen group were significantly lower than those of the SAH + vehicle group (*P* <0.05, Figure 4).

### Tamoxifen administration inhibited NF-κB DNA binding activity after subarachnoid hemorrhage

**Figure 5:**
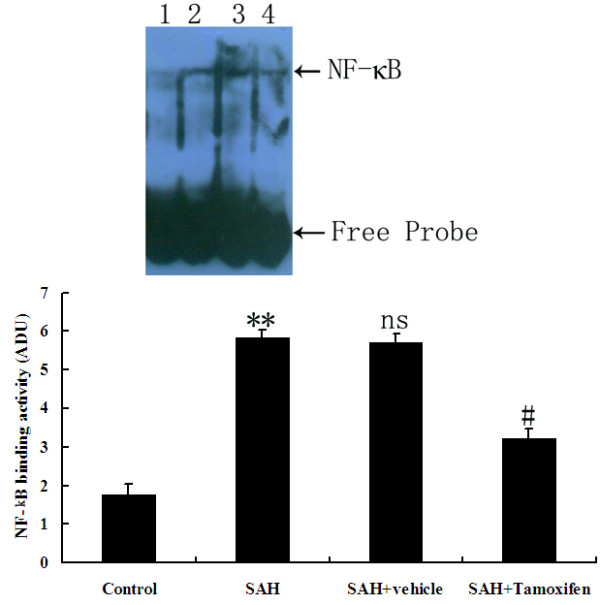
**NF-κB activity in the brain area surrounding the blood clot in control group (n = 6), subarachnoid hemorrhage (SAH) group (n = 6), SAH + vehicle group (n = 6), and SAH + tamoxifen group (n = 6).** Upper, electrophoretic mobility shift assay (EMSA) autoradiography of NF-κB DNA binding activity. Lane 1, control; lane 2, SAH; lane 3, SAH + vehicle; lane 4, SAH + tamoxifen, respectively. Bottom, Levels of NF-κB DNA binding activity quantified by computer-assisted densitometric scanning and expressed as arbitrary densitometric units (ADU). **P <0.01 between control animals versus SAH animals; #*P* <0.05 between SAH + vehicle animals versus SAH + tamoxifen animals; n.s. *P* >0.05 between SAH animals versus SAH + vehicle animals.

EMSA autoradiography of NF-κB DNA binding activity of the brain samples was shown in Figure 5. Low NF-κB binding activity (weak EMSA autoradiography) was found in the control group. Compared with control group, NF-κB binding activity in the injured brain was significantly increased (*P* <0.01) in SAH and vehicle-treated groups. In SAH + tamoxifen group, the NF-κB binding activity was significantly downregulated (*P* <0.05) in the brain area surrounding the blood clot site after SAH.

### Tamoxifen treatment decreased cortical levels of pro-inflammatory cytokines following subarachnoid hemorrhage

**Figure 6:**
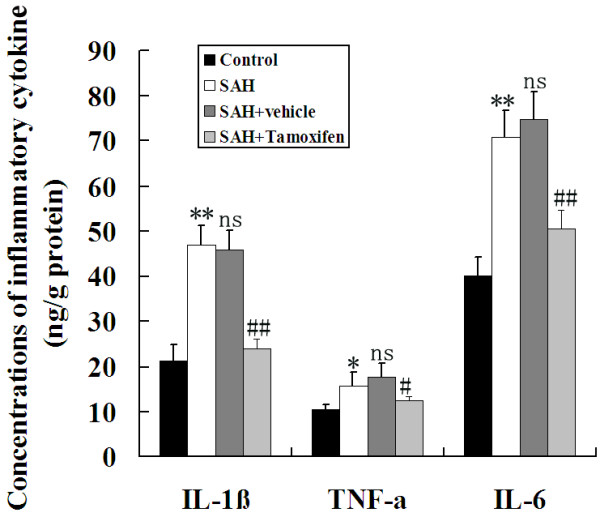
**Changes of inflammatory mediators in the injured brains as determined by ELISA in control group (n = 6), subarachnoid hemorrhage (SAH) group (n = 6), SAH + vehicle group (n = 6), and SAH + tamoxifen group (n = 6).** SAH could induce the significantly increased concentrations of IL-1β, TNF-α and IL-6 in the rat brain after SAH. In SAH + tamoxifen group, the cortical concentrations of IL-1β, TNF-α, and IL-6 were markedly downregulated following SAH. *P <0.05 and **P <0.01 between control animals versus SAH animals; #*P* <0.05 and ##*P* <0.01 between SAH + vehicle animals versus SAH + tamoxifen animals; n.s. *P* >0.05 between SAH animals versus SAH + vehicle animals.

Concentrations of IL-1β, TNF-α and IL-6 were low in the rat brains of control group (Figure 6). Compared with control group, cortical levels of the three inflammatory cytokines were greatly induced after SAH. As shown in Figure 6, tamoxifen administration after SAH could lead to significantly decreased IL-1β, TNF-α and IL-6 concentrations.

### Behavior testing

**Figure 7:**
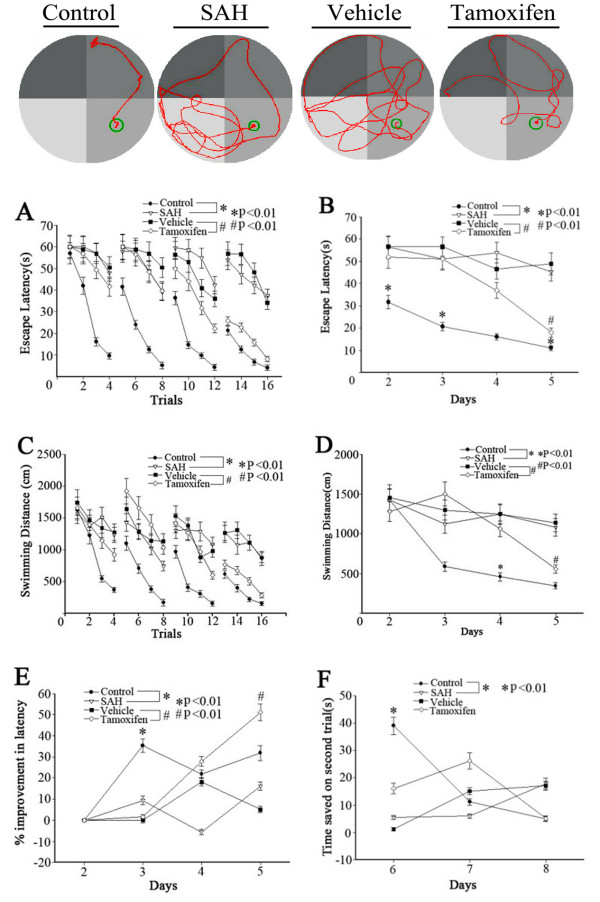
**Representative images of the Morris water maze (MWM) trials of the rats of four groups.** Bottom: Spatial learning and memory in the MWM. Escape latency and swimming distance over 16 trials **(A, C)** and averaged for each day **(B, D)** over days 2 to 5. The percent improvement in escape latency from the previous training day was significantly lower on day five in the subarachnoid hemorrhage (SAH) group as compared to controls (**E**, *P <0.01). The tamoxifen group was higher on day four than the vehicle group (**E**, #*P* <0.01). The control group exhibited significantly longer time except on day 6 as compared to the SAH group on the working memory task (matching-to-place task) by the time saved in latency for finding the platform on the second (test) trial compared to the first (sample) trial (**F**, *P <0.01). There was no significant difference between the tamoxifen group and the vehicle group (values are means ± SD, n = 10 per group).

The facility of MWM was shown in Figure 7, and one of the representative trials in each group was also indicated in Figure 7. For all behavioral measurements, swimming speed and thigmotaxis (percent time spent in the perimeter of the pool) were evaluated and found not significantly different among the four groups except for four rats with hemiparesis that spent significantly more time in the perimeter and showed swim-overs and jump-offs. These four animals were excluded from the present study.

Rats usually can find the visible platform. They usually found the platform on the first trial, even if they failed the trial, which happened equally across the groups. There was no difference in the escape latency and swimming speed in the cued learning procedure between the four groups (*P* >0.05). Spatial learning was also the same for all four groups during the second and third days after blood injection. Spatial learning deficits appeared during the fourth and fifth days in the SAH group as compared to controls. The tamoxifen group exhibited significantly shorter escape latency than the vehicle group during the fourth and fifth days (Figure 7A,B). Animals of all four groups learned to find the platform to escape from water within each testing day (Figure 7A-D). But the SAH group was significantly impaired compared to controls (the fourth and fifth days), which was alleviated by tamoxifen compared to the vehicle group. Repeated measures ANOVA indicated a significant difference in escape latency (Figure 7A, *P* <0.01) and in swimming distance (Figure 7C, *P* <0.01) between the SAH group and control groups, which was also improved by tamoxifen compared to the vehicle group. When the escape latency and swimming distance from all of the testing days was separated into the four daily trials, the escape latency of the SAH group was significantly longer compared to the control group. The tamoxifen group’s escape latency was significantly shorter than the vehicle group (Figure 7B, *P* <0.01). But in swimming distance, only the tamoxifen group performed significantly compared to the vehicle group on the 4th day (Figure 7D, *P* <0.01).

The percent improvement in latency of the SAH group decreased significantly by day five (Figure 7E, *P* <0.01), while rats in the control groups had gradual, non-significant decreases in percent improvements. However, the percent improvement of the tamoxifen group appeared as negative growth by day four. There was no significant difference between the groups in reference memory as measured by the percent time spent in the target quadrant in the probe trial, although there was a trend towards fewer annulus crossings between the four groups (data not shown). Animals in the control group showed significantly longer times except for the sixth day on the working memory task when compared to the SAH groups, as shown by the time saved in latency for finding the platform on the second trial compared to the first trial. However, there was no significant difference among the tamoxifen group and the vehicle group (Figure 7F, *P* >0.05).

### Effects of tamoxifen administration on control rats

**Figure 8:**
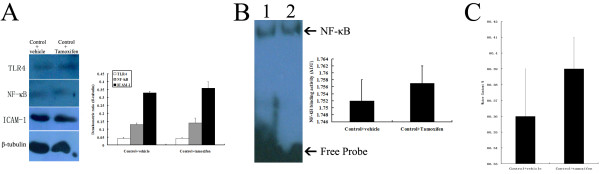
**No significant influence of tamoxifen on control rats. (A)** Western blot; **(B)** electrophoretic mobility shift assay (EMSA), lane 1, control + vehicle, lane 2, control + tamoxifen; **(C)** Water content analysis.

As shown in Figure 8, the protein levels of TLR4, NF-κB, and ICAM-1 were expressed at a low level in the rat brains of control + vehicle and control + tamoxifen groups and the difference was not significant (*P* >0.05). Compared with control + vehicle group, NF-κB binding activity in the brain was not significantly changed in control + tamoxifen group (*P* >0.05). The mean value of brain water content in the brain did not change significantly by tamoxifen administration in control rats (*P* >0.05).

## Discussion

The main findings of this study are as followings: 1) after tamoxifen administration, early brain damages such as brain edema, BBB permeability, and clinical scales were ameliorated after SAH; 2) the upregulated cortical levels of these agents related to the TLR4/NF-κB inflammatory signaling pathway were attenuated when treated with tamoxifen at the level of protein synthesis; 3) after intraperitoneal administration of tamoxifen, SAH-induced behavior and cognitive dysfunction were attenuated in this prechiasmatic blood injection model. These findings suggest for the first time that tamoxifen may attenuate the SAH-induced TLR4/NF-κB signaling pathway activation, which may facilitate the development of secondary brain damage following SAH. This neurobehavioral study leads to the hypothesis that decrease of inflammatory pathway in the brain may be important in modulating the function of cognition, and we tentatively suggest that this phenomenon may be involved in the mechanism of neurobehavioral dysfunction after SAH.

There have been several studies focusing on the neuroprotective effects of tamoxifen in other nerve injury models. Tian *et al*. [7] investigated whether tamoxifen could inhibit inflammatory response, reduce cell apoptosis, decrease myelin loss and ameliorate impaired behavior after adult rat spinal cord injury (SCI). Their results suggested that tamoxifen treatment attenuated post-traumatic inflammatory damage, alleviated neuronal apoptosis, decreased myelin loss and production in axonal outgrowth inhibitors, and resulted in improved functional outcome after SCI. In more recent data, Xie *et al*. [8] demonstrated in an intracerebral hemorrhage model that tamoxifen at 5 mg/kg reduced perihematomal brain edema, decreased caudate atrophy, and improved functional outcome. In this current research, our data are consistent with the previous studies. We found that tamoxifen administration following SAH could reduce cerebral edema, BBB permeability and neurologic scoring, which played important roles in EBI following SAH. At the same time, neurobehavioral results that are consistent with our speculation show that rats treated with tamoxifen exhibit a better performance in comparison to vehicle-treated rats in MWM testing, suggesting that downregulation of TLR4/NF-κB signaling may improve the SAH-induced spatial working memory dysfunction.

In the previous research regarding tamoxifen and inflammation, Chuang *et al*. [18] investigated the baseline levels of NF-κB activity of representative carcinoma cell lines, and the change of NF-κB activity in response to a challenge with tamoxifen treatment. They suggest that tamoxifen treatment significantly attenuated doxorubicin-induced NF-κB activation in the cells. Another research investigated whether tamoxifen alleviated inflammatory damage seen in the irradiated microglia *in vitro* and in the irradiated brain [19]. Tamoxifen administration (i.p., 5 mg/kg) immediately post-radiation reduced the irradiation-induced brain damage after whole brain irradiation (WBI), which included injuries of BBB permeability and tissue edema formation. Furthermore, tamoxifen downregulated WBI-induced microglial activation and reactive astrogliosis by attenuating the pro-inflammatory cytokine expression of IL-1β and TNF-α [19]. In the present study, we found that the levels of TLR4/ NF-κB pathway in the brain were activated following SAH and could be suppressed after tamoxifen administration, which is in agreement with the previous studies from other *in vivo* and *in vitro* models. However, the potential mechanism underlying the initial effect on TLR4/NF-κB signaling pathway following SAH remains unclear. The main TLR4 ligands in the brain after SAH and the whole mechanism related to tamoxifen call for further research.

Studies from basic research and clinical trial have demonstrated that inflammation played important roles in the pathophysiological process of SAH [20]. TLR4/NF-κB signaling pathway has also been proven to be activated in both injured brain tissue and spasmodic cerebral artery of SAH models in our previous studies [21,22]. Our present data proved that tamoxifen might repress the expressions of TLR4/NF-κB related inflammatory agents, which could lead to downregulation of cerebral inflammation after SAH. We tentatively suggest that due to the anti-inflammatory effect of tamoxifen administration, the learning and memory ability tested by cued learning procedure, spatial acquisition task, reference memory task, and working memory task were turned better following experimental SAH. Future research will be designed to look inside the mechanism of the whole signaling pathway of inflammatory response, to look for the key cell type in the brain after SAH, and to find out the exact organelles that were responsible for the transduction of the TLR4/NF-κB pathway. Previous studies concerning estrogen receptor modulators and inflammation indicated that glial cells (microglia and astroglia) might play an important role in this molecular mechanism [23,24]. Tamoxifen may be a candidate to counteract brain inflammation under neurodegenerative conditions by targeting the production and release of pro-inflammatory molecules by glial cells.

## Conclusions

To the best of our knowledge, this study is the first one to evaluate the effect of tamoxifen on the EBI and on secondary neurobehavioral dysfunction in this experimental SAH model, as well as the influence of tamoxifen on TLR4/NF-κB pro-inflammatory pathway after SAH. We found that SAH could upregulate the protein expressions of TLR4/NF-κB pathway-related mediators, upstream factors (TLR4 and NF-κB) and downstream factors (IL-1β, TNF-α, IL-6, and ICAM-1), in the brain surrounding the blood clot, which could be markedly inhibited by tamoxifen therapy. These results suggest that SAH could induce an activation of the TLR4/NF-κB signaling pathway in the rat brain, which might play a central role in the inflammatory response that leads to secondary insults after SAH. The therapeutic benefit of post-SAH tamoxifen administration might be due to its salutary effect on modulating the TLR4/NF-κB signaling pathway.

## Abbreviations

ADU: Arbitrary densitometric units; BBB: Blood-brain barrier; CNS: Central nervous system; EB: Evans blue; EBI: Early brain injury; ELISA: Enzyme-linked immunosorbent assay; EMSA: Electrophoretic mobility shift assay; ICAM-1: Intercellular adhesion molecule-1; IL-1β: Interleukin-1β; IL-6: Interleukin-6; i.p.: Intraperitoneally; LDF: Laser Doppler flow meter; MABP: Mean arterial blood pressure; MCA: Middle cerebral artery; MWM: Morris water maze; NF-κB: Nuclear factor-κB; SAH: Subarachnoid hemorrhage; TLR4: Toll-like receptor 4; TNF-α: Tumor necrosis factor-α.

## Competing interests

The authors declare that they have no competing interests.

## Authors’ contributions

ZW, GC, and XS performed all experimental studies and data acquisition, and contributed to the study conception, design, analysis, and data interpretation. GC and TH collected samples, performed data analysis, and drafted the manuscript. XS revised the manuscript. All authors read and approved the final manuscript.
